# Cognitive Behavioural Therapies for Weight-Loss in Adults: A Scoping Review Protocol

**DOI:** 10.3390/healthcare11182473

**Published:** 2023-09-06

**Authors:** Laura María Compañ-Gabucio, Diana Mancheño-Bañón, Laura Torres-Collado, Jesús Vioque, Manuela García-de-la-Hera

**Affiliations:** 1 Unidad de Epidemiología de la Nutrición (EPINUT), Departamento de Salud Pública, Historia de la Ciencia y Ginecología, Universidad Miguel Hernández (UMH), 03550 Alicante, Spain; lcompan@umh.es (L.M.C.-G.); dmancheno@umh.es (D.M.-B.); vioque@umh.es (J.V.); manoli@umh.es (M.G.-d.-l.-H.); 2 Instituto de Investigación Sanitaria y Biomédica de Alicante (ISABIAL), 03010 Alicante, Spain; 3 CIBER Epidemiología y Salud Pública (CIBERESP), Instituto de Salud Carlos III, 28034 Madrid, Spain

**Keywords:** excess body weight, weight loss, psychological intervention

## Abstract

Obesity and being overweight are very important public health issues due to their increasing prevalence worldwide. Third-wave cognitive behavioural therapies (3wCBT) have emerged in the last few years to promote weight loss. However, the scientific evidence identifying the most commonly used 3wCBT in weight-loss interventions in adults is still needed. The objective of this scoping review will be to identify the most widely researched 3wCBT used to facilitate weight loss in an adult population who are overweight and obese, according to the published scientific literature. The search will be carried out independently by two authors in PubMed (MEDLINE), Scopus, EMBASE, Web of Science, and PsycINFO, using search equations that contain keywords related to our search question: (1) population: adult and elderly population, (2) intervention: terms related to 3wCBT, and (3) results: weight loss or weight management. The data extraction will be performed following the indications of the Cochrane manual, and the results will be presented in three tables. The 3wCBTs have shown promising results for weight loss, but it is not yet known which of them is the most widely used to achieve weight loss in the adult population. Thus, the results of this scoping review could guide professionals in the psychological treatment of obesity and being overweight.

## 1. Introduction

The prevalence of obesity and being overweight has nearly doubled in the last decade, making them a significant public health issue [1]. In 2021, nearly 60% of adults suffered from being overweight or obese in the European Union [2]. These diseases are described as the major causes of premature mortality and morbidity worldwide [3]. They are also identified as major risk factors in the development of non-communicable diseases (NCDs) such as type 2 diabetes mellitus [4], non-alcoholic fatty liver disease [5], cardiovascular diseases (CVD) [6], neurodegenerative diseases [7], osteoarthritis [8], and several types of cancer [9]. These diseases can not only cause premature death but can also have a significant impact on psychosocial well-being and quality of life [10].

As previous studies have shown, overweight and obesity are complex multifactorial diseases that can be bidirectionally related to biological factors, environment, lifestyle, emotional and psychological factors [11]. Thus, an approach is needed that involves social support, medication, lifestyle interventions such as diet and physical activity, and even surgical interventions [12]. In this context, emerging weight management interventions are increasingly focusing on cognitive and emotional factors [13,14]. Third-wave cognitive behavioural therapies (3wCBT) stand out among these interventions as the most effective for weight loss in adults [15].

Hayes SC first named these therapies as “third wave” in 2004 [16], although they appeared in the late 1990s [17]. All three waves of therapy aim to modify behaviour [18]. The first wave, or behaviour therapy, is focused directly on problematic behaviour and emotion, based on conditioning principles such as reinforcing or discriminating [19]. The second wave, or cognitive–behavioural therapy, tries to modify behaviour through the restructuring of thoughts, applying an intrapersonal and cognitive approach. Finally, 3wCBT is also known as contextual therapy because its treatment involves context and its way of influencing behaviour [19]. In other words, 3wCBT combines strategies from first-wave therapies, which are more oriented to the history and circumstances of the person, and second-wave therapies, which are more focused on the cognitive component [20]. The 3wCBTs are focused on the relationship between people and their thoughts and the emotions they evoke, rather than the content of the thought itself [15]. For this purpose, 3wCBT emphasises aspects such as values, acceptance, mindfulness, objectives, goals, emotions, and metacognition [15]. However, not all interventions included in 3wCBT emphasise all these aspects, resulting in a wide range of therapies among which the following stand out: acceptance and commitment therapy, dialectical behaviour therapy, functional analytic psychotherapy, integrative behavioural couple therapy, compassion-focused therapy, and mindfulness-based cognitive therapy.

Currently, there are several published reviews on 3wCBT in patients who are overweight and/or obese [13,15,21,22,23,24,25,26,27]. Most of them are aimed at describing the effectiveness of one of the 3wCBTs on weight loss [22,23,24,27]. Sosa-Cordobés et al. [22] carried out a systematic review and meta-analysis aimed at determining the efficacy of mindfulness-based interventions for stress and weight reduction. This review included thirteen intervention studies in adults. The results of this review showed that mindfulness-based interventions were effective in reducing stress in the short term, but not in reducing weight or body mass index. Similarly, Ruffault et al. [27] found that mindfulness-based interventions had no significant effect on weight loss, but they did have a positive effect on physical activity levels. These results were based on twelve randomised intervention studies which were included in a systematic review with a meta-analysis aimed at conducting a comprehensive quantitative synthesis of the effects of mindfulness-based interventions on weight loss and health behaviours in adults who are overweight and obese. Iturbe et al. [23] also carried out a systematic review but it was focused on examining the effects of acceptance and commitment therapy on the weight management and psychological well-being of adults who are overweight or obese. Sixteen articles were included in this systematic review, half of which showed that acceptance and commitment therapy effectively addressed health behaviours related to weight control. The results found in the systematic review by Iturbe et al. are supported by those found in a very recently published review by Chew et al. [24]. These authors included thirteen studies in their recent systematic review and meta-analysis and showed that acceptance and commitment therapy was effective in achieving weight loss in terms of body mass index.

To a lesser extent, some of the reviews were focused on identifying which of the therapies included in 3wCBT is more effective for weight loss than standard psychological treatments [15,21,26]. Among the results described in these reviews, psychotherapy was found to be more effective for promoting weight loss in terms of body mass index than behavioural weight loss and/or health education [26]. In addition, cognitive behavioural therapy combined with a low-calorie diet was more effective in the promotion of weight loss in adults with obesity than a low-calorie diet alone [21]. Nevertheless moderate- to high-quality evidence suggested that 3wCBT led to a greater weight loss than standard behavioural treatment at post-intervention in adults who are overweight or obese [15]. Finally, another review described new ways of 3wCBT application in a weight-loss approach, for example, via smartphones [13], while another described how to use 3wCBTs to treat emotional eating, a condition which is closely related to being overweight and obesity [25].

The 3wCBTs promote and facilitate weight loss in people who are overweight and obese [15]. However, scientific evidence on which of the therapies included in 3wCBT is most commonly used in weight-loss interventions in adults is still unknown. Hence, we will conduct a scoping review to complement the existent scientific evidence by answering the following research question: According to the published scientific literature, which 3wCBT is used most frequently for weight-loss interventions in an adult population who are overweight or obese, and what does it consist of? Thus, the main objective of this scoping review will be to identify the most widely researched 3wCBT used to facilitate weight loss in an adult population who are overweight and obese, according to the published scientific literature.

## 2. Materials and Methods

This study has a scoping review protocol. Although this study is not a systematic review, we will follow the preferred Reporting Items for Systematic Reviews and Meta-Analysis (PRISMA) guidelines [28] and the PRISMA extension for Scoping Reviews (PRISMA-ScR) [29] in order to ensure both scientific rigor and that the content of this scoping review is complete and meets the expected standards. We will conduct a scoping review rather than a systematic review because our research question is broad and is not focused on determining the effectiveness, costs, or effect of a particular intervention [30].

### 2.1. Search Strategy

Based on the indications of previous articles [31], we will carry out a bibliographic search in four multidisciplinary databases, and one specific to psychology, in order to achieve a more complete search. According to these indications, an optimal combination of databases to cover the most published evidence is that composed of PubMed (MEDLINE), Scopus, EMBASE, and Web of Science, complemented by the search in a specific database in relation to our study interest, in this case, PsycINFO. Thus, we will carry out a bibliographic search in these five databases.

Each of these databases will be searched using the following equations, which will be created by combining search terms and the Boolean operators (AND and OR). The selected search terms describe our study population (adult, elder*), intervention (“third-wave”, “cognitive behavioural therapy”, mindfulness, “acceptance and commitment”, “dialectical behavior therapy”), and outcomes (“weight-loss”, “weight management”, “body weight”). We have the complete and outlined search equation to start the searches in the previously mentioned databases. The search equation will be used in each database in a staggered manner following the PICO structure, as shown in Table 1.

### 2.2. Eligibility Criteria

In order to be included in this scoping review, articles must meet the following criteria:

- Studies published in Spanish or English.
- Studies with an experimental design.
- Studies carried out in a population aged ≥18 years.
- Studies carried out in an adult population who are overweight or obese with or without other diseases (e.g., cardiovascular disease, metabolic syndrome, or others, but not eating disorders).
- Studies that explored an intervention for weight loss using at least one of the therapies included in 3wCBT (acceptance and commitment therapy, dialectical behaviour therapy, functional analytic psychotherapy, integrative behavioural couple therapy, compassion-focused therapy, mindfulness-based cognitive therapy).
- Studies in which weight loss was assessed through objective methods (questionnaires or anthropometric measures).
- Studies with available full text.

We will apply the exclusion criterion “full-text not available” when an article is not open access and we cannot obtain the full version either through our university library or after contacting the corresponding author of the article in question. We will not apply time filters or limitations of any kind in any of the databases that we consult, and we will apply all exclusion criteria manually.

### 2.3. Study Selection and Screening

For the selection of the studies, we will not use any bibliographic manager. We will only use the Microsoft Excel program version 16.0. Once the searches in the five databases have been carried out, we will download and unify all the titles of the articles found in an Excel sheet in which we will create a template with the exclusion criteria to facilitate the screening process. The first column will list all the titles, the second column will indicate whether each article is included (yes/no), and the third column will indicate the different exclusion criteria. Every time we indicate “no” in the inclusion of an article, we must mark the most obvious exclusion criteria in the template. This template will be prepared before carrying out the bibliographic search to avoid manipulation of the information and guarantee the transparency of the researchers during the screening process.

In this scoping review, two researchers (D.M.-B. and L.M.C.-G.) will carry out the selection of studies independently. The possible discrepancies that arise during this process in relation to the inclusion or exclusion of an article will be resolved by a third researcher (L.T.C.). The selection of studies will be carried out by removing duplicate articles and then conducting a screening process. Duplicate articles will be eliminated from the Excel database including all the titles that we find in the five above-mentioned databases. First, we will arrange the titles in alphabetical order, which will display titles beginning with symbols or special characters, such as brackets or parentheses, at the beginning of the Excel sheet. Second, we will remove these special symbols manually, that is to say, without applying the functionalities available for this purpose in Microsoft Excel. Third, we will arrange the titles in alphabetical order again in order to achieve a real alphabetical order of the titles. This will allow us to eliminate duplicates more easily, without needing to apply the direct commands available in Microsoft Excel for this purpose. Once we have eliminated the duplicated titles, we will apply the inclusion criteria to the remaining articles through a two-phase screening process. In the first phase of the screening process, the two researchers (D.M.-B. and L.M.C.-G.) will review the titles and abstracts, while in the second phase, they will review the full texts. A separate Excel sheet will be used for each screening phase. The screening process will be carried out carefully, discarding only those articles that we are sure do not meet the inclusion criteria. The study selection flow will be displayed graphically using the PRISMA diagram [32].

### 2.4. Data Extraction and Synthesis

The data will be extracted in three tables which will be prepared before beginning the bibliographic search to guarantee transparency and methodological rigor. Although this work is not a systematic review, we will follow the indications of the Cochrane manual [33] for the preparation of the tables. Following the manual’s recommendations, we will present the results in three tables, one showing the general characteristics of the included studies, another showing characteristics that are more specific to our research question, and the last one showing characteristics related to the quality and risk of bias of the included studies. The first table will include information regarding author/year, study design, sample/country, participants, intervention/comparator, assessment, and main study variables [34]. The second table will include author/year, participants and diagnosis, intervention, intervention duration, number of sessions, intervention manager, and main results [34]. The last table will include the author/year, main limitations, funding sources, and declarations of interest [35]. Two of the researchers (D.M.-B. and L.M.C.-G.) will be in charge of extracting the data in the three tables, and all the researchers will carry out the narrative synthesis of the information in the results section.

The synthesis of the extracted information will be carried out narratively, using tables and figures whenever possible. In the hypothetical case that the number of articles included is very high and the tables exceed the length of three pages (the maximum limit allowed in most scientific journals), we will create a database including the items from each table and will perform a univariate descriptive analysis using the free software R version 4.0.4. In this same hypothetical scenario, we will perform a qualitative content analysis on the variables in which the variety of information is so great that we are unable to use quantitative methods of graphical presentation, such as pie charts or bar charts. The qualitative analysis will be carried out using the WordCloud package available in the free software R. WordCloud is a package that allows us to analyse the discourse and represent it graphically through word maps with different colours. This package assigns the same colour to words that are repeated the same number of times. Those words that are repeated more often will appear in a larger size than the rest of the words.

### 2.5. Quality Assessment

We do not plan to assess the quality of the articles to be included critically and objectively through tools or questionnaires. The quality assessment of the articles to be included is a mandatory requirement for systematic reviews but not for scoping reviews [36]. However, in order to alert readers to the quality of the articles included, we will provide a specific table on the risk of bias indicators closely related to the quality of the articles, such as conflicts of interest, funding, and limitations of each of the articles included. In addition, we will include a specific heading: “Main limitations and risk of bias of included studies” in the results section in order to describe possible low-quality indicators.

### 2.6. Protocol Registration

This protocol was registered in the Open Science Framework (OSF) on 7 June 2023 and is available from: osf.io/yd8av [37].

## 3. Results

**Figure 1:**
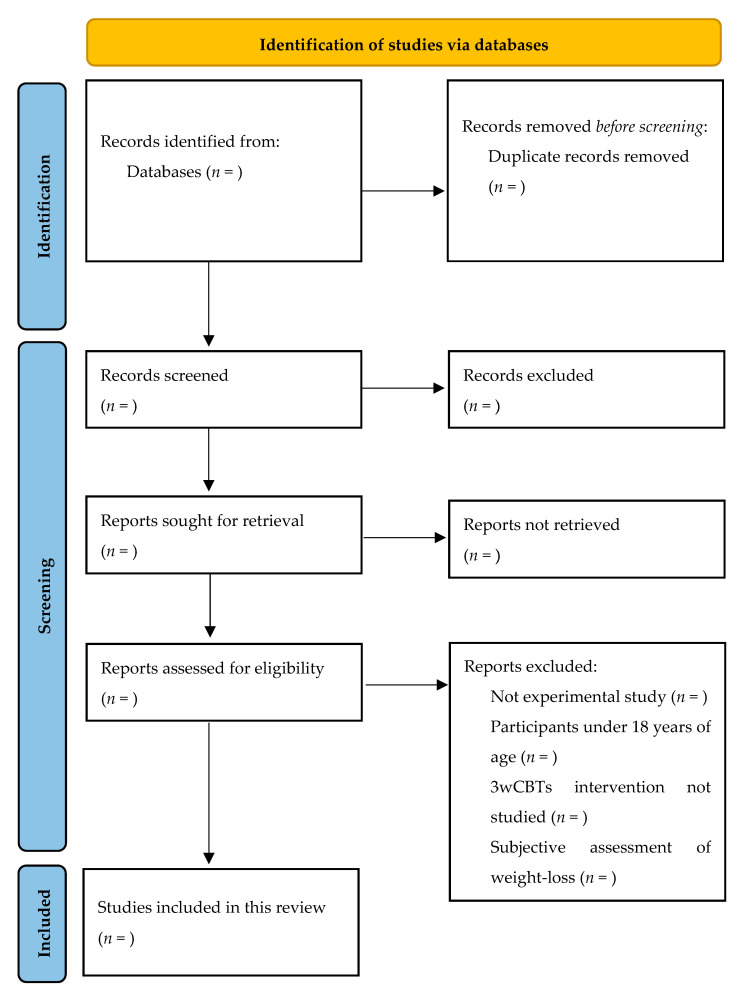
PRISMA flowchart to be used.

In this section of results, we expect to include information from randomised and non-randomised clinical trials on the characteristics of the 3wCBT most used to facilitate weight loss in the adult population who are overweight or obese. The study selection and screening will be presented in a PRISMA flowchart (Figure 1). We will carry out the synthesis of the studies included in this review in both narrative and tabular forms. Results will be reported in accordance with the Cochrane manual in order to ensure methodological rigor. Once completed, the results of this scoping review will be submitted to a peer-reviewed journal indexed in the Journal Citations Reports (JCR).

## 4. Discussion

As pointed out in the introduction section, the increase in the prevalence of obesity and being overweight has been accompanied by an increase in non-communicable diseases [38] and, consequently, a rise in premature deaths and increased healthcare costs [39]. Obesity and being overweight are characterised by high morbidity which results in the need for costly multifactorial treatment [40]. Effective strategies to deal with the negative consequences of obesity and being overweight should take biological, social, economic, political, and psychological aspects into account [11]. Interventions to promote weight loss are usually based on physical activity and diet recommendations, but they only appear to be effective in the short term [41,42]. In this sense, previous evidence suggested that 50% of individuals who received either a combined physical activity and diet intervention or only a diet intervention regained weight after the intervention [43].

In this context, emergent weight management interventions are increasingly treating psychological and emotional factors and have been associated with long-term weight loss [44]. Weight loss can be influenced by different emotional and psychological factors [45,46,47,48], such as a lack of weight-loss objectives, eating disinhibition, binge eating, deficient self-monitoring in healthy habits such as dietary intake or weight control, poor coping strategies in moments of distress, low motivation, external locus of control (attitudes that reflect a lack of commitment to taking responsibility for one’s own problems), low self-esteem, and depression or anxiety. Thus, a behavioural approach to obesity and being overweight should be considered.

The 3wCBTs address some of the abovementioned psychological issues through an emphasis on the acceptance rather than avoidance of uncomfortable internal sensations, such as food cravings, and a commitment to making behavioural choices that are in line with personal values and goals [20]. Key aspects of 3wCBT include elements of mindfulness and acceptance that are not included in traditional behavioural weight-loss interventions [49]. A recently published meta-analysis concluded that 3wCBT weight-loss interventions produced greater post-intervention weight loss, even after a 24-month follow-up, than standard behavioural weight-loss therapies [15]. Scientific evidence not only attributes effectiveness for weight reduction in adults with obesity and/or being overweight to 3wCBTs in general but also to each of the different therapies that constitute it.

Dialectical behaviour therapy is especially effective in the treatment of adults who are overweight/obese and experience emotional eating given its focus on the regulation of emotions [50]. A randomised intervention study showed that dialectical behaviour therapy was helpful in reducing weight in overweight/obese women with binge eating disorders [51]. Another intervention study showed a weight loss of 3 kg in adults who are overweight/obese and experience emotional eating and who were treated with dialectical behaviour therapy [52]. Mindfulness-based cognitive therapy, on the other hand, seeks to improve the ability to be fully aware of the present experience, which has been linked to a positive impact on changing the eating behaviours of obese populations [53]. In this sense, a recent meta-analysis on weight-loss mindfulness-based cognitive therapy showed a significant positive effect of these interventions on emotional eating and a moderate effect on weight loss [54]. Compassion-focused therapy is focused on reducing self-criticism and shame, two aspects that can act as a trigger in adults who are overweight/obese [55] and, consequently, increase the risk of depression and anxiety which can hinder weight loss. Some evidence suggests that compassion-focused therapy interventions are promising for achieving weight loss and improving nutritional behaviours [56], as well as for reducing binge eating [57] and body weight shame [58]. In the weight-management context, acceptance and commitment therapy could improve the ability to cope with the negative effects of food cravings and the ability to engage in healthy behaviours that are consistent with one’s values [59]. Scientific evidence has shown that acceptance and commitment therapy is one of the most effective 3wCBTs for weight loss in the adult population [15] and, in particular, for the reduction in body mass index [24]. In contrast, according to some authors, one of the most difficult 3wCBTs to research is functional analytic psychotherapy [60]. This is an approach based on the behavioural principle of natural and social reinforcement within a genuine and authentic psychotherapeutic relationship whose main target is a social connection [61]. Improvements in social connection using this approach can be useful in treating women with lipoedema, a chronic progressive adipose disorder [62].

A large body of scientific evidence supports the beneficial effects of 3wCBT on weight reduction. These interventions, both in general and individually, appear to be effective for weight loss in overweight/obese adults. Much of this evidence is provided by review papers, although none of them have addressed the objective of identifying which of the 3wCBTs is the most used in weight-loss intervention studies. Therefore, we consider that this scoping review may be of interest to the scientific community and can provide it with a current synthesis of the characteristics of the 3wCBT that has been most studied in intervention research. Thus, carrying out this scoping review could, therefore, facilitate the development of evidence-based interventions for weight loss in overweight/obese adults.

### Limitations

Our scoping review has certain limitations that may influence our results. Although we sought to carry out a systematic peer review to ensure scientific rigor, the possible lack of information reported in some studies, the publication bias which limits the publication of the null results of the interventions, and the selection bias are limitations for the majority of reviews. First, we excluded articles that were not written in English or Spanish, which could lead to the loss of potentially important information, increasing the selection bias. However, articles on important 3wCBTs such as mindfulness and acceptance-based treatments are mostly (>90%) written in English [63]. Second, we excluded all articles whose full text we could not access. This can also increase the selection bias, however, we tried to obtain the full text through our university library services or by contacting the corresponding author. 

The selection bias could also be increased by the fact that we only searched in five databases so some important articles from other databases could have been overlooked, although these five databases are the most comprehensive and recommended for literature searches in reviews [31]. The third limitation of this article is that we only included experimental studies that had a small sample size or other biases associated with this type of study. In addition, we did not assess the quality of the included studies because this is not a mandatory requirement for scoping reviews. As a result, it is possible that some low-quality articles could have been included in our review. To deal with this limitation, we included a table with characteristics related to the quality and risk of bias of the included articles to ensure the transparency of the results obtained. In any case, this limitation should not alter the results of this scoping review as our aim was to describe the characteristics of the most commonly used 3wCBT interventions to promote weight loss, but not their effectiveness. Based on our previous experience with scoping reviews, the fourth limitation we possibly experienced resulted from the difficulty in synthesising the results. This is because we usually encounter a great deal of variation in some of the characteristics of the included studies, such as assessment tools, study outcomes, and duration and description of the interventions studied. Finally, it was difficult to establish the search strategy because the classification of 3wCBT varies according to the published articles. However, in our search strategy, we included the most used 3wCBTs found in similar published reviews.

This scoping review also has some strengths. It provided a description of a wide variety of 3wCBTs that can be used in weight management, offering professionals the opportunity to learn about a therapy that could be considered optimal for their patients. In addition, this scoping review is novel and provides an overview of the experimental studies existing on 3wCBT weight-loss intervention. Finally, we want to point out that, as is the case in most scoping reviews, we had the opportunity to identify different knowledge gaps in this topic [64] from which future research can emerge.

## 5. Conclusions

Being overweight and obesity have relevant negative consequences on the health of the adult population, including increased premature mortality. Therefore, the study of different interventions to address these conditions is sorely needed. The use of 3wCBTs has shown promising results for weight loss, but it is not yet known which of them is the most widely used to achieve weight loss in the adult population. Thus, it was pertinent to carry out this scoping review.

## Figures and Tables

**Table 1:** Search strategy.

Population	#1	adult OR elder*
Intervention	#2	“third-wave” OR cognitive behavioural therapy” OR mindfulness OR “acceptance and commitment” OR “dialectical behavior therapy”
Comparison or control	NA	NA
Outcome	#3	“weight-loss” OR “weight management” OR “body weight”
Complete	#1 AND #2 AND #3	

NA, not applicable.

## Data Availability

The data presented in this study are available on request from the corresponding author.
